# Extra View: Sirt1 Acts As A Gatekeeper Of Replication Initiation To Preserve Genomic Stability.

**DOI:** 10.1080/19491034.2018.1456218

**Published:** 2018-04-20

**Authors:** Koichi Utani, Mirit I. Aladjem

**Affiliations:** a Department of Microbiology, Kanazawa Medical University, Uchinada Ishikawa, Japan; b Developmental Therapeutics Branch, Center for Cancer Research, NCI, NIH, Bethesda, MD, USA

**Keywords:** Cell cycle, Chromatin, Chromosome, DNA replication, Epigenetics

## Abstract

Since the discovery of a yeast gene silencing modifier (Silent Information Modifier 2, SIR2) and its role in maintaining genomic stability more than two decades ago, SIR2 homologs (sirtuins) were identified in diverse species. Sirtuins are protein deacetylases that play diverse roles in proper cellular metabolism including cell cycle progression and maintenance of genomic stability. In yeast, SIR2 interacts with replication origins and protein complexes that affect both replication origin usage and gene silencing. In metazoans, the largest SIR2 homolog, SIRT1, is implicated in epigenetic modifications, circadian signaling, DNA recombination and DNA repair. Until recently, very few studies investigated the role of mammalian SIRT1 in modulating DNA replication. We discuss a newly characterized interaction between human SIRT1 and the DNA replication machinery, reviewing data from recent studies that have investigated how complex signaling pathways that involve SIRT1 affect cellular growth regulatory circuits.

## Introduction

Two decades ago, the yeast protein silent information regulator 2 (SIR2) was identified as a gene silencing modifier essential for proper cell cycle progression, radiation resistance and genomic stability [1]. Since then, SIR2 homologs (sirtuins) were identified in diverse species, including a family of genes encompassing seven NAD+ dependent deacetylases (SIRT1-7) in humans. SIRT1 is the largest and the most intensely investigated sirtuin because its activation extends life span, modulates senescence and mediates the effects of caloric restriction in organisms from yeast to humans [2]. In yeast, SIR2 interacts with replication origins and protein complexes that regulate DNA replication and transcription, modulating both replication origin usage and gene silencing [3]. In metazoans, SIRT1 is a nuclear protein that can deacetylate multiple target proteins [4] implicated in epigenetic modifications, circadian signaling, DNA recombination and DNA repair.

SIRT1 affects numerous nuclear processes. The link between SIRT1 and genomic stability was first observed in yeast. Either loss or suppression of yeast SIR2 leads to chromosome instability and cellular senescence [5]. Cellular senescence associated with SIR2 deletion is accompanied with the accumulation of circular rDNA due to increased recombination activity [6], similar to the phenotype observed in yeast with a deletion of *sgs1,* the human homologue of BLM helicase [7]. SIRT1 in metazoans modulates various nuclear processes, including affecting cellular sensitivity to DNA-damaging agents, the efficiency of DNA repair and heterochromatin formation. When SIRT1 is depleted in mice, the majority of SIRT1 depleted mice exhibit embryonic lethality. Fibroblasts established from SIRT1 depleted embryos are impaired in DNA damage response and repair pathways [8]. The aberrant chromatin modifications in SIRT1 depleted fibroblasts are accompanied by chromosomal instability [8,9], and the phenotypes of conditional, tissue-specific *SIRT1* deletion mutants implicate SIRT1 activity in the response to oxidative stress during embryonic hematopoiesis and tumorigenesis [10].

Until recently, very few studies explored the role of mammalian SIRT1 in modulating DNA replication. Below, we discuss a newly characterized interaction between human SIRT1 and the DNA replication machinery, reviewing data from recent studies that have investigated how complex signaling pathways that involve SIRT1 affect cellular growth regulatory circuits.

## SIRT1 substrates in the mammalian nucleus

Potential SIRT1 substrates can be identified by finding proteins that share the SIRT1 recognition motif, bind overexpressed SIRT1 or exhibit elevated levels of acetylation in SIRT1-depleted cells. Of the 1598 potential SIRT1 substrates in the human acetylome [11,12], the most abundant SIRT1 histone substrate is lysine 16 of histone H4, a marker of active transcription. Histone deacetylation is of particular significance in the modulation of gene expression, as it can potentially alter nucleosome structure and can often affect transcription [13].

In addition to histones, SIRT1 is known to deacetylate various non-histone proteins implicated in transcription. For example, SIRT1 deacetylate lysine 310 of RelA/p65, the nuclear cleaved form of NF-kB that regulates NF-kB transcription activity and p53-dependent apoptosis [14], and CREB binding protein (CBP), which is deacetylated to modulate CREB-dependent transcription. SIRT1 substrates also include components of the AMP-activated protein kinase (AMPK)-mediated cAMP and phosphorylation sensing cascade, including AMPK, forkhead box O transcriptional factors (FOXOs), the transcription coactivator PCG1alpha and the transcription factor HIF1alpha [14,15].

One group of SIRT1 substrates regulates circadian rhythms, signaling networks which maintain 24-hour periodicities in diverse species from cyanobacteria to higher eukaryotes. The core activities that regulate circadian rhythms, although mediated by non-homologous proteins, are highly conserved in evolution [16]. In metazoans, circadian oscillation is mediated by the transcriptionally balanced feedback loop between the BMAL1-CLOCK protein acetyl transferase [16] and the transcription regulators PER1-3, CRY1 and CRY2 [17]. SIRT1 interacts directly with BMAL1-CLOCK, removing CLOCK-mediated acetylation of histone H3-Lys9 (H3K9), histone H3-Lys4 (H3K4), and BMAL1 Lys537. SIRT1 also deacetylates PER2, enhancing its degradation and creating a negative feedback loop affecting the oscillation [18,19]. Another SIRT1 substrate that negatively regulates BMAL-CLOCK is the TIMELESS protein, which is also a replisome complex member [20]. TIMELESS regulates MYC-mediated G1/S transition and WEE1-mediated G2 checkpoints [21]. In actively replicated cells, TIMELESS dissociates from the replisome with peroxiredoxin when levels of reactive oxygen species increase [22], thereby slowing replication and linking the SIRT1-regulated circadian rhythm circuitry with the pace of DNA replication and key cell cycle transitions.

A group of SIRT1 substrates regulate various aspects of the DNA damage response. SIRT1 promotes DNA damage signaling by deacetylating NBS1 [23], homologous recombination by deacetylating the WRN helicase [24], non-homologous end-joining by deacetylating Ku70 [25], base excision repair by deacetylating the APE1 endonuclease [26] and nucleotide excision repair by deacetylating XPA and XPC [27,28]. In addition, SIRT1 activates p53 through deacetylation of Lys382 [29], countering acetylation by p300/CBP. SIRT1-mediated deacetylation of p53 results in its degradation by ubiquitination, thereby protecting the cells from p53-induced apoptosis [30,31]. SIRT1 also deacetylates the ubiquitin ligase MDM2 at K182 and K185 resulting in its degradation and protection of p53 from ubiquitin-mediated degradation [32]. By regulating p53, SIRT1 determines cell fate after DNA damage. In addition to its role in DNA damage signaling, SIRT1 acts as an oxidative stress sensor, as oxidative stress introduces reversible oxidative modifications of SIRT1 cysteines, leading to diminished deacetylation activity [33].

## SIRT1-mediated regulation of DNA replication

Chromosome duplication in eukaryotes necessitates the assembly of a protein complex (pre-replication complex, or pre-RC) on DNA sequences termed replication origins. In yeast, protein-DNA interactions that lead to DNA replication depend partly on specific DNA sequences, as exemplified by recruitment of the pre-RC on replication origins, also named autonomously replicating sequence (ARS) elements [34]. Yeast SIR2 binds some ARS elements and represses replication initiation from these replication origins by creating an inhibitory chromatin structure that silences both transcription and replication [3].

In a similar manner, mammalian SIRT1 interacts with host factors to create a repressive chromatin environment that curtails the replication of some dsDNA viruses. Although many dsDNA viruses encode their own replication proteins [35], their replication can be affected by the host cell's epigenetic modifications that are modulated by SIRT1. For example, either SIRT1 depletion or the SIRT1 inhibitor EX-527 can impair the propagation of human papillomaviruses by abolishing recruitment of ATM, NBS1 and Rad51 to the genome [36]. Specifically, HPV16 E1-E2 replication complexes directly interact with SIRT1 at the viral replication origin, and SIRT1 depletion enhances viral replication, concomitant with increased acetylation and stabilization of the E2 protein [37]. Hepatitis B virus minichromosomes also bind SIRT1 concomitant with HDAC1 and acetyltransferase CBP/p300 [38]. Finally, SIRT1 is a key silencer of the reactivation of Kaposi's sarcoma-associated herpesvirus, suppressing the transcription of the master lytic transactivator RTA [39].

SIRT1 interacts with the mammalian replication initiation machinery. SIRT1 interacts with MCM10 [40], which activates replication by enabling DNA unwinding, and with TopBP1 [41,42], which activates the ATR kinase important for replication stress signaling. SIRT1 depletion leads to reduced fork velocity, increased origin firing and impaired intra-S-phase checkpoint whereby cells continue to synthesize DNA after exposure to DNA damage [8,41]. Consistent with these findings, recent acetylome studies in both human and murine cells have identified many replication associated proteins that are potential SIRT1 substrates, such as CDK6, MCMs, TIMELESS, RepID/PHIP and LIG1 [11] [12].

Human chromosomes contain many potential replication origins, but chromosome duplication only initiates at a fraction of those potential origins each cell cycle. Although human replication origins tend to share several chromatin features, they do not exhibit a clear consensus sequence, suggesting that replication can initiate at diverse sequence motifs and are activated intermittently, often in a tissue-specific manner [43]. The excess potential origins can be activated to initiate replication in particular tissues or under specific conditions, such as replication stress or conditions that slow replication fork elongation [44,45]. The activation of excess origins, which normally remain dormant, can be genotoxic if over-replicated DNA accumulates [46,47].

**Figure 1.**
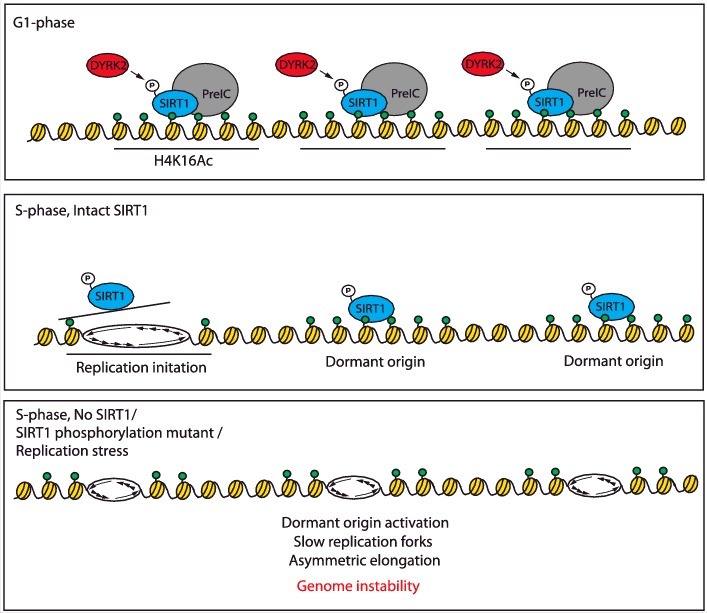
Interactions of SIRT1 with active and dormant replication origins.

We have recently shown that SIRT1 substrate H4K16AC, as well as a form of SIRT1 phosphorylated on threonine 530 (T530-pSIRT1), bind both active and dormant replication origins [48]. T530-pSIRT1-bound dormant origins can be induced to initiate replication by exposing cells to low doses of aphidicolin, a DNA polymerase inhibitor. These dormant origins were also activated in cells in which SIRT1 was depleted and in cells containing a form of SIRT1 harboring an alanine substitution mutation in T530, preventing phosphorylation. Although T530-pSIRT1 was found to bind origins and interact with a group of replication-associated proteins including MCM2, DDK, ORC2, RPA1 and PCNA, it neither interacted nor colocalized with elongating replication forks. In concordance, SIRT1 and EdU, a thymidine analog, did not colocalize in nuclei of replicating mammalian cells during the S-phase [48]. These results are consistent with the observed absence of SIRT1 in samples of chromatin obtained by precipitation of EdU-labeled replicating DNA (iPOND) [49] and suggest that T530-pSIRT1 interacts with the replication initiation machinery to prevent excess initiation of DNA replication (Figure 1).

## Is there a role for SIRT1-regulated replication origins in genome maintenance?

The evidence for a role of SIRT1 in regulating replication initiation frequency suggests that SIRT1, and especially the T530-phosphorylated form of SIRT1, plays a role in maintenance of genomic stability. The link between a functional, phosphorylatable SIRT1 and genomic stability is evident from the increased abundance of extrachromosomal elements and micronuclei observed when cells with no SIRT1 or cells harboring the non-phosphorylatable mutant of SIRT1 are exposed to mild perturbation of DNA replication [48]. However, although phosphorylation of SIRT1 facilitates its deacetylation activity and H4K16 acetylation is a hallmark of replication origins, it is yet unclear whether deacetylation is directly required for suppression of dormant origins and regulation of extrachromosomal elements.

Genomic stability is also affected by the crosstalk between SIRT1 and PARP1, either directly or via utilization of the same NAD+ substrate [19]. SIRT1 also regulates the acetylation of pRb and H4K16, modulating the interactions of those substrates with telomeric regions associated with aging and senescence and critically supporting genome maintenance in cells with a deficiency in the parallel pathway involving BRCA1^50^. SIRT1-mediated genome maintenance is also evident through modulation of the DNA damage response: acetylation of H4K16 is required for H3K36me3-mediated recruitment of this histone to double-stranded DNA breaks and for prevention of aberrant accumulation of the protein 53bp1 in the chromatin of cells deficient in components of the Fanconi anemia pathway [51]. The transcriptional consequences of histone acetylation and the differential DNA repair capacity of open and condensed chromatin might indicate SIRT1 could modulate DNA repair via additional mechanisms.

Context-dependent alterations of chromatin by SIRT1 may underlie the observation that manipulation of SIRT1 levels can exert both protective and stimulatory effects on cancer development. For example, heterozygous and homozygous deletions of *SIRT1* have opposite effects on colon cancer formation in mice [52]. In another example, in cells with misregulated SIRT1 and BRCA1 haploinsufficiency [50] and in cells with impaired TIP60 [51, an increased association of acetylated pRb and acetylated H4K16 with chromatin increases the dependence of cells on homologous recombination to prevent senescence and genomic instability. The effects of SIRT1 on replication dynamics and genomic stability might therefore reflect pathways that affect replication indirectly by creating chromatin environments that alter DNA repair pathway choices.

SIRT1 phosphorylation on T530 can be mediated by two kinases: DYRK2 and JNK1. Human DYRK2, which phosphorylates numerous substrates including c-Jun and Myc, acts as a negative regulator of G1/S transition, as shown by the shortening of the G1 phase in cells depleted of DYRK2 [53]. The association of p530-SIRT1 with replication origins and the resulting suppression of initiation in a group of those origins are consistent with the notion that DYRK2-mediated phosphorylation regulates the pace of progression through the cell cycle, preventing early initiation of excess replication origins during S-phase. Over-initiation, both during normal growth and under replicative stress, which can be induced by overexpression of some oncogenes, has dire consequences. These consequences include inappropriate checkpoint activation [54], depletion of nucleotides and RPA and discoordination between transcription and replication [55]. Further, over-replication can deplete nucleotide pools and decrease replication fork speed, resulting in stalled replication forks and DNA breaks [56,57]. The observation that binding of T530-pSIRT1 to chromatin at potential replication origins can prevent over-replication is consistent with the identification of DYRK2 in a screen for genes that affect chromosomal stability [58].

DYRK2 forms a complex with the EDVP E3 ligase (EDD/UBR5, DDB1 and VprBP/DCAF1 [59]), which is involved in diverse cell cycle regulatory interactions including G2 arrest. The Vpr (human immunodeficiency virus type 1 viral protein R) component of EDVP disrupts nuclear membranes to arrest cells at the G2 phase of the cell cycle, allowing virions to escape the nucleus [60]. Vpr mediated nuclear disruption is regulated by WEE-1 [61] and can be triggered via the ATR-gamma-H2AX, RPA32 and 53BP1 axis in response to DNA damage or replication stress [62]. In addition, Vpr enhances MCM10 polyubiquitylation and degradation by interacting with DDB1-Cul4A-VprBP E3 ligase to divert host replication at G2/M arrest [63]. The interaction of DYRK2 with a ubiquitin ligase is of interest, as components of Cul4A-anchored ubiquitin ligases are known to affect the initiation of DNA replication. For example, RepID/DCAF14, a member of the CRL4 ubiquitin ligase complex, binds and activates a group of replication origins [64]. This interaction strongly suggests that ubiquitin ligases, perhaps modified by DYRK2 and its substrates, are involved in the regulation of DNA replication. In addition, the indirect association between DYRK2 with MCM10, a replicative helicase accessory factor, is in line with the notion that DYRK2 plays a role in the regulation of replication origin choice via SIRT1 phosphorylation.

Another kinase that can potentially catalyze the phosphorylation of T530 on SIRT1 is JNK1. The interaction between JNK1 and SIRT1 was identified under conditions of oxidative stress. JNK1 phosphorylates SIRT1 on three sites, Ser27, Ser47 and Thr530, ehnacing the nuclear localization and enzymatic activity of SIRT1 [65]. JNK1-mediated phosphorylation might protect cells from prematurely activating too many origins upon mild stress that could be addressed without massive activation of dormant origins. The associations between over-initiation and DNA damage are reflected in the observation that overexpression of oncogenes preferentially leads to DNA damage at fragile sites [56,66]. The direct interactions we observe at replication origins, which specifically bind p530-SIRT1, suggest that SIRT1 phosphorylation modifies the local chromatin environment to fine-tune the frequency of replication initiation and thereby protect cells from the consequence of aberrant replication.

## Conclusions

Post-translational protein modifications play critical roles in modulating chromatin transactions, and recent observations increasingly imply that the NAD^+^-dependent SIRT1 deacetylase links cellular metabolic activity and external signaling pathways with cell proliferation, stress responses and genome stability. Previous analyses have shown that sirtuins modulate the acetylation status of chromatin modifiers, including histones, transcription factors, chromatin remodeling complexes and components of the circadian rhythm circuitry. SIRT1-mediated chromatin modifications can create restrictive chromatin environments and limit the activation of distinct signaling networks. Recent observations in mammalian cells suggest that in addition to this indirect role, SIRT1 interacts directly with the DNA replication machinery. SIRT1 modifies protein components of pre-initiation complexes and binds chromatin directly to limit initiation from a subgroup of replication origins. SIRT1 origin binding activities require the phosphorylation of SIRT1 by kinases that are essential for genomic stability, providing another possible link between the cellular environment and chromatin modification. These observations propose a novel, SIRT1-mediated regulatory pathway that modulates replication patterns to insure proper cellular response to replication stress.
